# Supplementary material for: Ocular surface manifestations of coronavirus disease 2019 (COVID-19): A systematic review and meta-analysis
Source: PLoS One. 2020 Nov 5;15(11):e0241661. doi: 10.1371/journal.pone.0241661 (PMC7643964; doi:10.1371/journal.pone.0241661)
Supplement: S2 Appendix — (DOCX) [file pone.0241661.s002.docx]

**S2 Appendix.**

**History**

[Download history](https://www.ncbi.nlm.nih.gov/pubmed?p$l=Email&Mode=download&dlid=history&filename=history.csv&db=pubmed&historyid=NCID_1_162531733_130.14.18.48_5555_1588480932_567933400_0MetA0_S_HStore&p$debugoutput=off)[Clear history](https://www.ncbi.nlm.nih.gov/pubmed/advanced)

| Recent queries | | | | |
| --- | --- | --- | --- | --- |
| Search | Add to builder | Query | Items found | Time |
| [#33](https://www.ncbi.nlm.nih.gov/pubmed/advanced) | [Add](https://www.ncbi.nlm.nih.gov/pubmed/advanced) | Search **((((((((((((conjunctiva) OR "Conjunctiva"[Mesh]) OR ((((((((Conjunctivas) OR Tunica Conjunctiva) OR Palpebral Conjunctiva) OR Conjunctiva, Palpebral) OR Bulbar Conjunctiva) OR Conjunctiva, Bulbar) OR Plica Semilunaris of Conjunctiva) OR Plicae Semilunares of Conjunctiva)) OR Ocular manifestations) OR "Eye Manifestations"[Mesh]) OR "Ophthalmic Manifestations") OR (“Ocular Infections, Viral” or “Infection, Viral Ocular” or “Infections, Viral Ocular” or “Ocular Infection, Viral” or “Viral Ocular Infection” or “Viral Ocular Infections” or “Viral Eye Infections” Eye Infection, Viral” or “Infection, Viral Eye” or “Infections, Viral Eye” or “Viral Eye Infection”)) OR "Eye Infections"[Mesh]) OR "Conjunctivitis"[Mesh]) OR "Conjunctivitis, Viral"[Mesh]) OR viral conjunctivitis)) AND ((((((((((((covid 19) OR coronavirus covid-19) OR coronavirus) OR novel coronavirus) OR wuhan coronavirus) OR corona virus) OR coronavirus infection))) OR novel coronavirus pneumonia) OR new coronavirus) OR coronavirus china)** | 52 | 01:31:41 |
| [#32](https://www.ncbi.nlm.nih.gov/pubmed/advanced) | [Add](https://www.ncbi.nlm.nih.gov/pubmed/advanced) | Search **(((((((((((covid 19) OR coronavirus covid-19) OR coronavirus) OR novel coronavirus) OR wuhan coronavirus) OR corona virus) OR coronavirus infection))) OR novel coronavirus pneumonia) OR new coronavirus) OR coronavirus china** | [27073](https://www.ncbi.nlm.nih.gov/pubmed/?cmd=HistorySearch&querykey=32) | 01:26:10 |
| [#31](https://www.ncbi.nlm.nih.gov/pubmed/advanced) | [Add](https://www.ncbi.nlm.nih.gov/pubmed/advanced) | Search **((((((((((conjunctiva) OR "Conjunctiva"[Mesh]) OR ((((((((Conjunctivas) OR Tunica Conjunctiva) OR Palpebral Conjunctiva) OR Conjunctiva, Palpebral) OR Bulbar Conjunctiva) OR Conjunctiva, Bulbar) OR Plica Semilunaris of Conjunctiva) OR Plicae Semilunares of Conjunctiva)) OR Ocular manifestations) OR "Eye Manifestations"[Mesh]) OR "Ophthalmic Manifestations") OR (“Ocular Infections, Viral” or “Infection, Viral Ocular” or “Infections, Viral Ocular” or “Ocular Infection, Viral” or “Viral Ocular Infection” or “Viral Ocular Infections” or “Viral Eye Infections” Eye Infection, Viral” or “Infection, Viral Eye” or “Infections, Viral Eye” or “Viral Eye Infection”)) OR "Eye Infections"[Mesh]) OR "Conjunctivitis"[Mesh]) OR "Conjunctivitis, Viral"[Mesh]) OR viral conjunctivitis** | [35529](https://www.ncbi.nlm.nih.gov/pubmed/?cmd=HistorySearch&querykey=31) | 01:25:28 |
| [#28](https://www.ncbi.nlm.nih.gov/pubmed/advanced) | [Add](https://www.ncbi.nlm.nih.gov/pubmed/advanced) | Search **viral conjunctivitis** | [1668](https://www.ncbi.nlm.nih.gov/pubmed/?cmd=HistorySearch&querykey=28) | 01:24:01 |
| [#30](https://www.ncbi.nlm.nih.gov/pubmed/advanced) | [Add](https://www.ncbi.nlm.nih.gov/pubmed/advanced) | Search **"Conjunctivitis, Viral"[Mesh]** | [736](https://www.ncbi.nlm.nih.gov/pubmed/?cmd=HistorySearch&querykey=30) | 01:18:59 |
| [#27](https://www.ncbi.nlm.nih.gov/pubmed/advanced) | [Add](https://www.ncbi.nlm.nih.gov/pubmed/advanced) | Search **"Conjunctivitis"[Mesh]** | [18981](https://www.ncbi.nlm.nih.gov/pubmed/?cmd=HistorySearch&querykey=27) | 01:18:21 |
| [#24](https://www.ncbi.nlm.nih.gov/pubmed/advanced) | [Add](https://www.ncbi.nlm.nih.gov/pubmed/advanced) | Search **"Eye Infections"[Mesh]** | [36843](https://www.ncbi.nlm.nih.gov/pubmed/?cmd=HistorySearch&querykey=24) | 01:11:28 |
| [#21](https://www.ncbi.nlm.nih.gov/pubmed/advanced) | [Add](https://www.ncbi.nlm.nih.gov/pubmed/advanced) | Search **“Ocular Infections, Viral” or “Infection, Viral Ocular” or “Infections, Viral Ocular” or “Ocular Infection, Viral” or “Viral Ocular Infection” or “Viral Ocular Infections” or “Viral Eye Infections” Eye Infection, Viral” or “Infection, Viral Eye” or “Infections, Viral Eye” or “Viral Eye Infection”** | [3073](https://www.ncbi.nlm.nih.gov/pubmed/?cmd=HistorySearch&querykey=21) | 01:09:12 |
| [#19](https://www.ncbi.nlm.nih.gov/pubmed/advanced) | [Add](https://www.ncbi.nlm.nih.gov/pubmed/advanced) | Search **"Eye Infections, Viral"[Mesh]** | [8873](https://www.ncbi.nlm.nih.gov/pubmed/?cmd=HistorySearch&querykey=19) | 01:05:01 |
| [#15](https://www.ncbi.nlm.nih.gov/pubmed/advanced) | [Add](https://www.ncbi.nlm.nih.gov/pubmed/advanced) | Search **"Ophthalmic Manifestations"** | [685](https://www.ncbi.nlm.nih.gov/pubmed/?cmd=HistorySearch&querykey=15) | 00:59:28 |
| [#10](https://www.ncbi.nlm.nih.gov/pubmed/advanced) | [Add](https://www.ncbi.nlm.nih.gov/pubmed/advanced) | Search **"Eye Manifestations"[Mesh]** | [4943](https://www.ncbi.nlm.nih.gov/pubmed/?cmd=HistorySearch&querykey=10) | 00:48:16 |
| [#6](https://www.ncbi.nlm.nih.gov/pubmed/advanced) | [Add](https://www.ncbi.nlm.nih.gov/pubmed/advanced) | Search **Ocular manifestations** | [7846](https://www.ncbi.nlm.nih.gov/pubmed/?cmd=HistorySearch&querykey=6) | 00:46:21 |
| [#5](https://www.ncbi.nlm.nih.gov/pubmed/advanced) | [Add](https://www.ncbi.nlm.nih.gov/pubmed/advanced) | Search **(((((((Conjunctivas) OR Tunica Conjunctiva) OR Palpebral Conjunctiva) OR Conjunctiva, Palpebral) OR Bulbar Conjunctiva) OR Conjunctiva, Bulbar) OR Plica Semilunaris of Conjunctiva) OR Plicae Semilunares of Conjunctiva** | [22208](https://www.ncbi.nlm.nih.gov/pubmed/?cmd=HistorySearch&querykey=5) | 00:45:34 |
| [#4](https://www.ncbi.nlm.nih.gov/pubmed/advanced) | [Add](https://www.ncbi.nlm.nih.gov/pubmed/advanced) | Search **"Conjunctiva"[Mesh]** | [15391](https://www.ncbi.nlm.nih.gov/pubmed/?cmd=HistorySearch&querykey=4) | 00:42:52 |
| [#1](https://www.ncbi.nlm.nih.gov/pubmed/advanced) | [Add](https://www.ncbi.nlm.nih.gov/pubmed/advanced) | Search **conjunctiva** | [22150](https://www.ncbi.nlm.nih.gov/pubmed/?cmd=HistorySearch&querykey=1) | 00:42:12 |
