# Supplementary material for: Ocular surface manifestations of coronavirus disease 2019 (COVID-19): A systematic review and meta-analysis
Source: PLoS One. 2020 Nov 5;15(11):e0241661. doi: 10.1371/journal.pone.0241661 (PMC7643964; doi:10.1371/journal.pone.0241661)
Supplement: Attachment — Submitted filename: Dr. Nandi_Plos Occular manifestation of COVID-19 review_comments.docx [file pone.0241661.s003.docx]

**Ocular surface manifestations of coronavirus disease 2019 (COVID-19): a systematic review and meta-analysis**

**Reviewer’s comments**

In this study, the authors have described the meta-analysis of cases having ocular manifestation of COVID-19 around the world. The manuscript highlights the meta-analysis of clinical symptoms during ocular manifestation of COVID-19 such as ocular pain, discharge, redness and follicular conjunctivitis. The meta-analysis has been performed in which 222 citations were identified by online database search. After screening process described by the authors, 16 papers were found relevant. The data from 16 research articles was used which were published from China, Netherlands, Iran, Singapore, Italy and Brazil. The data includes a sum total of 2347 positive cases out of 3064 patients. Out of these, 196 patients (8.35%) were reported with ocular manifestation.

The meta-analysis does not include any studies published from India.

Also, the meta-analysis of pooled data revealed that 6.91% of the patients with ocular manifestations suffered from severe pneumonia the association of ocular manifestation with the severity of the respiratory complications or the prognosis is not discussed in details.

Incidence refers to the occurrence of new cases of disease or injury in a population over a specified period of time.

**Abstract: Prevalence:** Prevalence in epidemiology is the proportion of a particular population found to be affected by a medical condition at a specific time.

**Incidence** refers to the occurrence of new cases of disease or injury in a population over a specified period of time.

But the population included in this study is not uniform. So instead of prevalence, **incidence/occurrence** can be a better word.

- The study states that 11.6% patients had ocular manifestation of COVID-19. Out of these Ocular pain (31.2%), discharge (19.2%), redness (10.8%), and follicular conjunctivitis (7.7%) were the main features. But 31.2 + 19.2 + 10.8 + 7.7 = 68.9 %.
- Does remaining 31.1% patients signify some other uncommon symptoms? Please specify in details.

**Introduction**

- The studies published thus far suffer from significant publication bias and heterogeneity, and robust data collection and reporting of ocular manifestations is needed in the future. (Please re-phrase this sentence)
- It was declared a public health emergency of international concern on 30th January 2020 and in March 2020, the World Health Organization (WHO) declared the COVID-19 outbreak a pandemic (Please rephrase this sentence: Outbreak and Pandemic are two different epidemiological terms).
- The causative pathogen of this potentially fatal disease is a novel enveloped RNA beta coronavirus 2, a member of the Coronaviridae family, also known as severe acute respiratory syndrome coronavirus2 (Please rephrase this sentence. Enveloped positive sense ss-RNA virus. *Betacoronavirus* and *Coronaviridae* is written in Italics)
- During the current pandemic, there have been various reports of ocular involvement including features of follicular conjunctivitis in patients infected with SARS-CoV 2 with some of them even demonstrating the presence of viral RNA in conjunctival or tear specimens **~~collected from these patients~~.**
- Rate of reverse transcriptase polymerase chain reaction (RT-PCR) positivity for viral RNA in ocular fluids (The rate of positivity can be calculated in a Cohort study. Because rate is with respect to time in which the study is been performed. In a cross-sectional study, only number of positive cases can be stated)

**Materials and methods**

- Confirmed COVID-19 cases indicate patients who were **diagnosed COVID-19 positive either on the basis of clinical criteria** or positive RT-PCR for viral RNA from nasopharyngeal swabs (Please check about this line). (The diagnosis should be done on the basis of clinical signs and confirmation by RT-PCR. Only clinical criteria might not be sufficient)
- The heterogeneity on the basis of I^2^:

• 0% to 40%: might not be important;

• 30% to 60%: may represent moderate heterogeneity;

• 50% to 90%: may represent substantial heterogeneity;

• 75% to 100%: considerable heterogeneity.

Please provide a reference it might be needed.

**Study outcome:**

- Clinical features, demographic profile and ocular complications of COVID-19 patients with ophthalmic manifestation (Ocular and ophthalmic is repetition: might need to rephrase the sentence)

**Risk of bias:** The risk of bias is demonstrated to be moderate in this study. But it needs more elaboration. Also, in the discussion part questions have been raised by the authors regarding risk of bias. This might contradict the study.

Table 1:

- The reference Zhang et al (Ref no 24) is actually Xu et al., 2020 (Please check author names)
- (**Reference number should be checked properly: The reference numbers given in the table are interchanged**)
- Tostmann et al: (Ref no 21): 20 number
- Zhou et al: (Ref no 22): 21 number
- Qianqian et al., (Ref no 23) is actually Lan et al., 2020
- Table 1: row number 7 reference not cited properly

**Discussion**

Several questions have been raised in the discussion part of the review. Possibilities of various bias that can be encountered during the data collections have been discussed such as recall bias, study design bias and bias due to pre-existing ocular features. Apart from this, few more constraints such as in severe cases ocular features may go unnoticed or non-specific conditions such as dry eyes, pain chemosis and redness. These conditions might subject the judgement of healthcare workers to be biased. Therefore, in many cases the conjunctival swabs or tear samples did not test positive for SARS-CoV-2 RNA by qRT-PCR.

But the data presented in this study shows, only 2.2% of the patients show ocular manifestation as the first clinical sign. Apart from this 6.9% patients with ocular symptoms suffered from severe pneumonia. This data is not sufficient to have a concrete conclusion. More data analysis might be considered for the analysis. Also, the analysis should be supported by significant statistical analysis.
