# Supplementary material for: Ocular surface manifestations of coronavirus disease 2019 (COVID-19): A systematic review and meta-analysis
Source: PLoS One. 2020 Nov 5;15(11):e0241661. doi: 10.1371/journal.pone.0241661 (PMC7643964; doi:10.1371/journal.pone.0241661)
Supplement: Attachment — Submitted filename: Dr. Nandis comments PlosOne.docx [file pone.0241661.s005.docx]

**Title: Ocular surface manifestations of coronavirus disease 2019 (COVID-19): a systematic review and meta-analysis**

The title is adequate for the content, informative, concise and clear

In this study, the authors have described the meta-analysis of cases having ocular manifestation of COVID-19 around the world. The manuscript highlights the meta-analysis of clinical symptoms during ocular manifestation of COVID-19 such as ocular pain, discharge, redness and follicular conjunctivitis.

**Abstract**: The abstract is comprehensive by itself. The important and essential information of the article has been included. The queries asked have been answered effectively.

**References**: Some minor changes suggested in the reference section have been corrected by the authors. Appropriate and adequate references related to the work are covered sufficiently covered in the list.

**Figures and tables**: The tables have been corrected according to the changes suggested previously. The information given in the table is necessary and very well presented in the tables and figures. The legends for figures and tables are self-explanatory.

**English**: The English used in the article is readable and good enough to convey the scientific meaning correctly.

**Recommendations:** The authors have adequately addressed the comments previously made. All the queries are answered satisfactorily. **The article should be accepted for publication.**
